# Re-analysis of whole-exome sequencing data reveals a novel splicing variant in the *SLC2A1* in a patient with GLUT1 Deficiency Syndrome 1 accompanied by hemangioma: a case report

**DOI:** 10.1186/s12920-021-01045-3

**Published:** 2021-07-31

**Authors:** Tugce Bozkurt, Yasemin Alanay, Ugur Isik, Ugur Sezerman

**Affiliations:** 1 Biostatistics and Bioinformatics Program, Graduate School of Health Sciences, Acibadem Mehmet Ali Aydinlar University, Istanbul, Turkey; 2 Division of Pediatric Genetics, Department of Pediatrics, School of Medicine, Acibadem Mehmet Ali Aydinlar University, Istanbul, Turkey; 3 Division of Pediatric Neurology, Department of Pediatrics, School of Medicine, Acibadem Mehmet Ali Aydinlar University, Istanbul, Turkey

**Keywords:** Whole exome sequencing, *SLC2A1*, GLUT1 Deficiency Syndrome 1, Ketogenic diet, Hemangioma

## Abstract

**Background:**

GLUT1 Deficiency Syndrome 1 (GLUT1DS1) is a neurological disorder caused by either heterozygous or homozygous mutations in the Solute Carrier Family 2, Member 1 (*SLC2A1*) gene. *SLC2A1* encodes Glucose transporter type 1 (GLUT1) protein, which is the primary glucose transporter at the blood–brain barrier. A ketogenic diet (KD) provides an alternative fuel for brain metabolism to treat impaired glucose transport. By reanalyzing exome data, we identified a de novo heterozygous *SLC2A1* variant in a girl with epilepsy. After reversed phenotyping with neurometabolic tests, she was diagnosed with GLUT1DS1 and started on a KD. The patient's symptoms responded to the diet. Here, we report a patient with GLUT1DS1 with a novel *SLC2A1* mutation. She also has a hemangioma which has not been reported in association with this syndrome before.

**Case presentation:**

A 5-year 8-month girl with global developmental delay, spasticity, intellectual disability, dysarthric speech, abnormal eye movements, and hemangioma. The electroencephalography (EEG) result revealed that she had epilepsy. Magnetic resonance imaging (MRI) showed that non-specific white matter abnormalities. Whole Exome Sequencing (WES) was previously performed, but the case remained unsolved. The re-analysis of WES data revealed a heterozygous splicing variant in the *SLC2A1* gene. Segregation analysis with parental DNA samples indicated that the variant occurred de novo. Lumbar puncture (LP) confirmed the diagnosis, and the patient started on a KD. Her seizures responded to the KD. She has been seizure-free since shortly after the initiation of the diet. She also had decreased involuntary movements, her speech became more understandable, and her vocabulary increased after the diet.

**Conclusions:**

We identified a novel de novo variant in the *SLC2A1* gene in a patient who previously had a negative WES result. The patient has been diagnosed with GLUT1DS1. The syndrome is a treatable condition, but the differential diagnosis is not an easy process due to showing a wide range of phenotypic spectrum and the overlapping symptoms with other neurological diseases. The diagnosis necessitates a genomic testing approach. Our findings also highlight the importance of re-analysis to undiagnosed cases after initial WES to reveal disease-causing variants.

## Background

The Solute Carrier Family 2, Member 1 gene (*SLC2A1*, OMIM *138140) is located on the 1p34.2 and encodes Glucose transporter type 1 (GLUT1) composed of 492 amino acids [1, 2]. GLUT1 is the primary membrane transporter responsible for the delivery of glucose in human erythrocytes and blood-tissue barriers [3–5]. Impaired glucose transport at the blood–brain barrier caused by mutations in the *SLC2A1* result in GLUT1 Deficiency Syndrome 1 (GLUT1DS1, OMIM #606777), which is a rare neurometabolic disorder [6, 7]. In 1991, De Vivo et al. firstly identified GLUT1DS1 in the two patients with low glucose concentrations in cerebrospinal fluid (CSF)-termed hypoglycorrhachia, seizures, delayed development, dysarthria, acquired microcephaly, and movement disorder including spasticity, ataxia, and dystonia. Seven years after this report, Seidner et al. revealed that mutations in the *SLC2A1* gene are the genetic factor behind the GLUT1DS1 [8].

The current diagnosis approaches are generally based on the molecular analysis of the *SLC2A1* gene and lumbar puncture for CSF evaluation [9] since hypoglycorrhachia is the hallmark of the syndrome [6]. The CSF/blood glucose ratio is usually less than 0.4 and CSF glucose level should be less than 60 mg/dl for the diagnosis [10]. Another sign for the syndrome is the *SLC2A1* mutations which predominantly occur de novo with the autosomal dominant condition [11]. In familial cases, mutations are usually inherited by autosomal dominant pattern; yet, autosomal recessive variants have also been shown [12–15]. Thus far, it is estimated that more than 140 different pathogenic variants have been reported in the *SLC2A1* [16]. Despite these reports, the relationship between phenotype and genotype for the syndrome could not be clarified thoroughly [17]. However, missense mutations can be associated with the whole symptom spectrum of GLUT1DS1, while deletions in the *SLC2A1* are related to more severe forms of the syndrome. [18]. Furthermore, the phenotypic range of GLUT1DS has been broadened in recent years [19]. All these facts make a precise diagnosis for this syndrome a challenging task for clinicians. On the other hand, it is vital to identify GLUT1DS1 in the early stages since the ketogenic diet (KD) therapy has the potential to improve symptoms [20].

## Case presentation

### Clinical Phenotype

The patient was a 5-year 8-month-old girl at the time of presentation, with epilepsy, intellectual disability, and a movement disorder. She was born to a 29-year-old woman, G3P1, at term, with c- section. She had extreme crying at the age of 2.5 months. She also had left lower eyelid hemangioma at birth. She was diagnosed with epilepsy at the age of 4 months. She had head control at the age of 1 year; she sat at 3 years; she never walked without assistance. She had delayed speech. She had microcephaly (46.5 cm, < 3 p); she has a dysarthric speech. Her tonus was increased in all four extremities; she also had involuntary movements (choreoathetosis and dystonia). Her deep tendon reflexes were increased in the lower extremities. She could only walk with assistance. Her 3 T magnetic resonance imaging (MRI) showed non-specific millimetric white matter hyperintensities. Her EEG showed left temporoparietal and right posterior temporal interictal epileptiform discharges that also generalized. The patient was on Levetiracetam for seizures. Lumbar puncture (LP) was performed to confirm the genetic finding. LP showed no cells, protein 20,40 mg/dl, glucose 32 mg/dl, simultaneous blood glucose 87 mg/dl (before the lumbar puncture, after a 4-h fasting), and a negative CSF culture.

### Whole exome sequencing data analysis

The quality of raw reads was checked using FastQC (v0.11.8) [21]. Skewer (v0.2.2) [22] was used to remove adapters and low quality (Phred quality score < 20) bases. Trimmed reads were aligned to the reference human genome (hg19) using the Burrows-Wheeler Aligner (BWA mem, v0.7.17) [23]. Post-alignment processing was performed following the Genome Analysis Toolkit’s (GATK, v4.0.11.0) [24] best practice recommendations. Picard tool package (v1.141) [25] was used to mark duplicate reads and fix the information of mate reads. Haplotyping and joint genotyping were also performed following GATK recommendation. Annovar (v2018-12-04) [26] was used to annotate each variant by getting biologically significant information from several databases.

### Variant filtration strategy

**Fig. 1:**
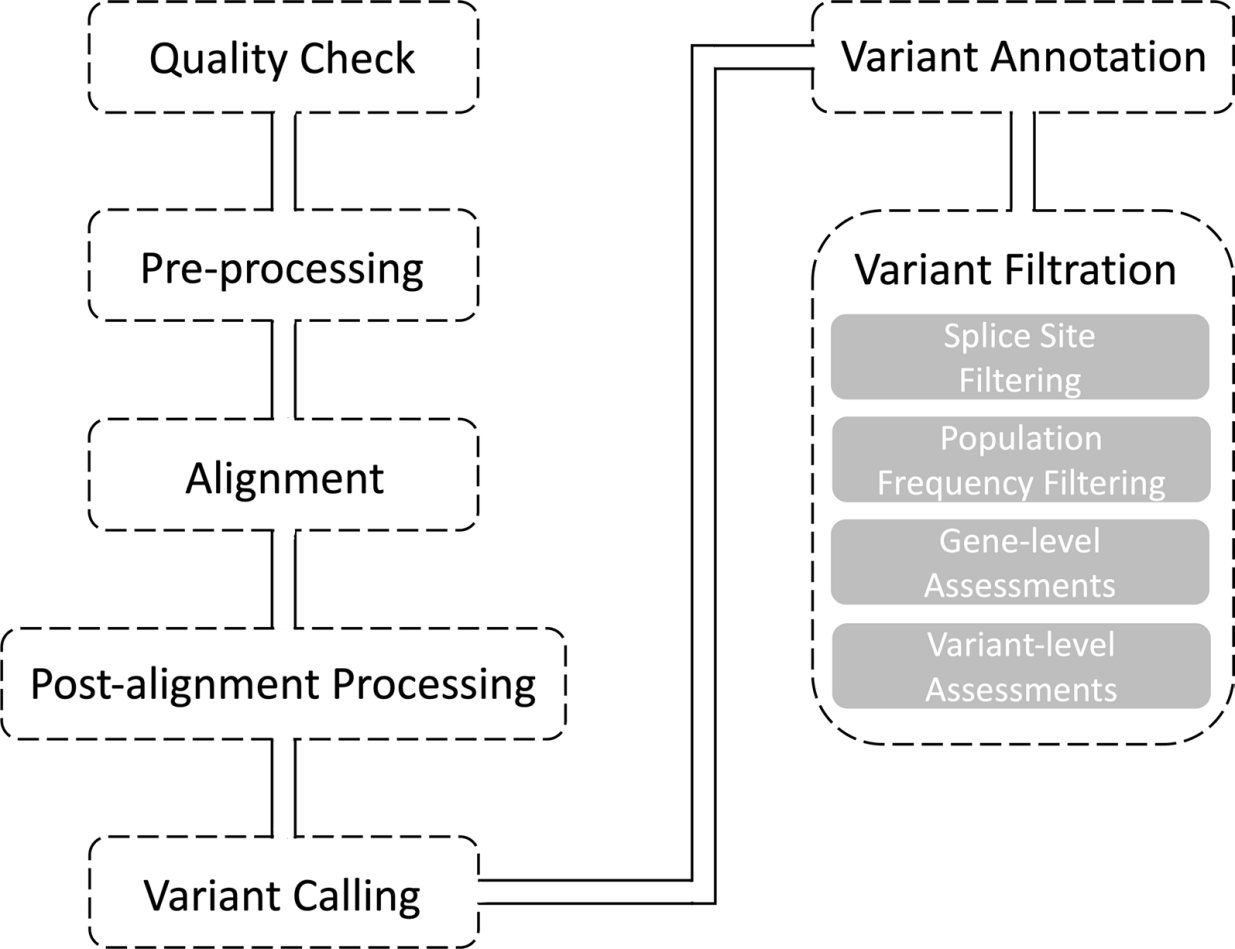
Bioinformatics workflow

After WES data analysis, 10,800 heterozygous and 5,500 homozygous variants passed the depth and quality filter. A phenotype-driven prioritization strategy was employed to detected variants. According to the mode of inheritance, variant filtering was performed by either absence-approach that assumes that disease-causing variants are not found in healthy population databases or setting the minor allele frequency threshold at < 0.1% [27]. The variants that are more than ten bases away from the exon–intron boundaries were excluded to filter the splicing variants. After the population frequency and splice site filtering, the number of variants decreased to 402 and 15, respectively. Several gene-level assessments, including the genic intolerance score [28] and gnomAD metrics [29], and variant level assessments such as CADD [30], REVEL [31], M-CAP [32] were utilized to interpret the pathogenicity. Then, the variants were prioritized based on the symptoms of the affected individual by collecting evidence from various sources; Online Mendelian Inheritance in Man (OMIM) [33], Mouse Genome Informatics (MGI) [34], and literature search. The variants were visualized via the Integrative Genomics Viewer (IGV) [35] to coverage check. The whole bioinformatics workflow was shown in Fig. 1.

**Fig. 2:**
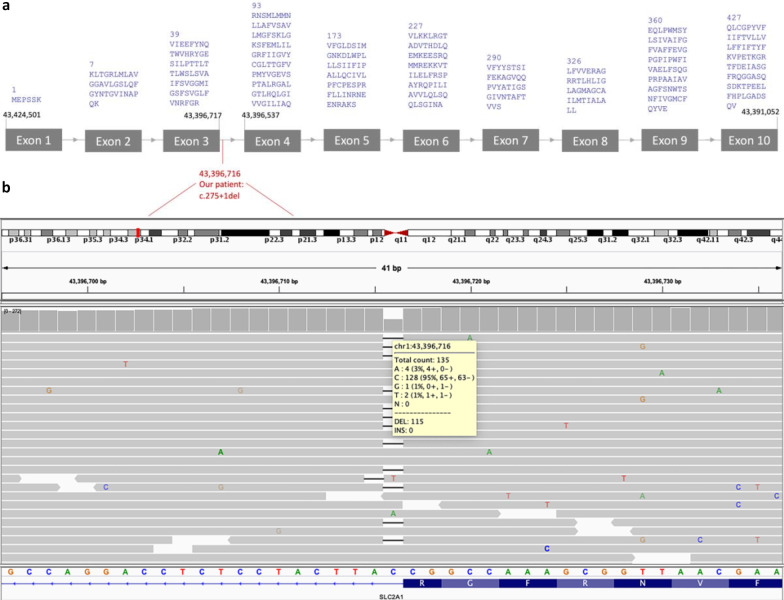
Schematic representation of SLC2A1 mRNA transcript (NM_6516.4) and IGV visualization of the variant. **a** SLC2A1 gene has 10 exons that encode GLUT1 composed of 492 amino acids. **b** The patient has variant c.275 + 1 in the heterozygous state. There are 128 reads for reference base **c** and 115 deletions at the position of 43,396,716 in the IGV visualization

Combining all of these computational evaluations provided strong evidence that the novel variant in the *SLC2A1* gene causes the observed phenotype. The heterozygous mutation in *SLC2A1* (NM_006516), c.275 + 1del classified as “Uncertain significance” based on the 2015 guidelines of the American College of Medical Genetics and Genomics (ACMG) [36]. The mutation was detected with high-depth reading in IGV (Fig. 2).

### Parental segregation analysis

Since the patient was a proband-only WES case, Sanger sequencing was carried out for parental segregation analysis. Peripheral blood samples were collected from the patient and her biological parents following the standard procedures. Genomic DNA was isolated from peripheral blood samples using the Quick-DNA™ Miniprep Plus Kit (Zymo Research, USA) following the manufacturer’s protocol. DNA quality was checked by agarose gel electrophoresis and NanoDrop 2000c Spectrophotometer (Thermo Fisher Scientific, USA). Sanger sequencing was carried out using standard protocols. PCR conditions and primer sequences can be provided upon request. Sequence electropherograms were visualized using the 4Peaks (Mekentosj, Amsterdam).

**Fig. 3:**
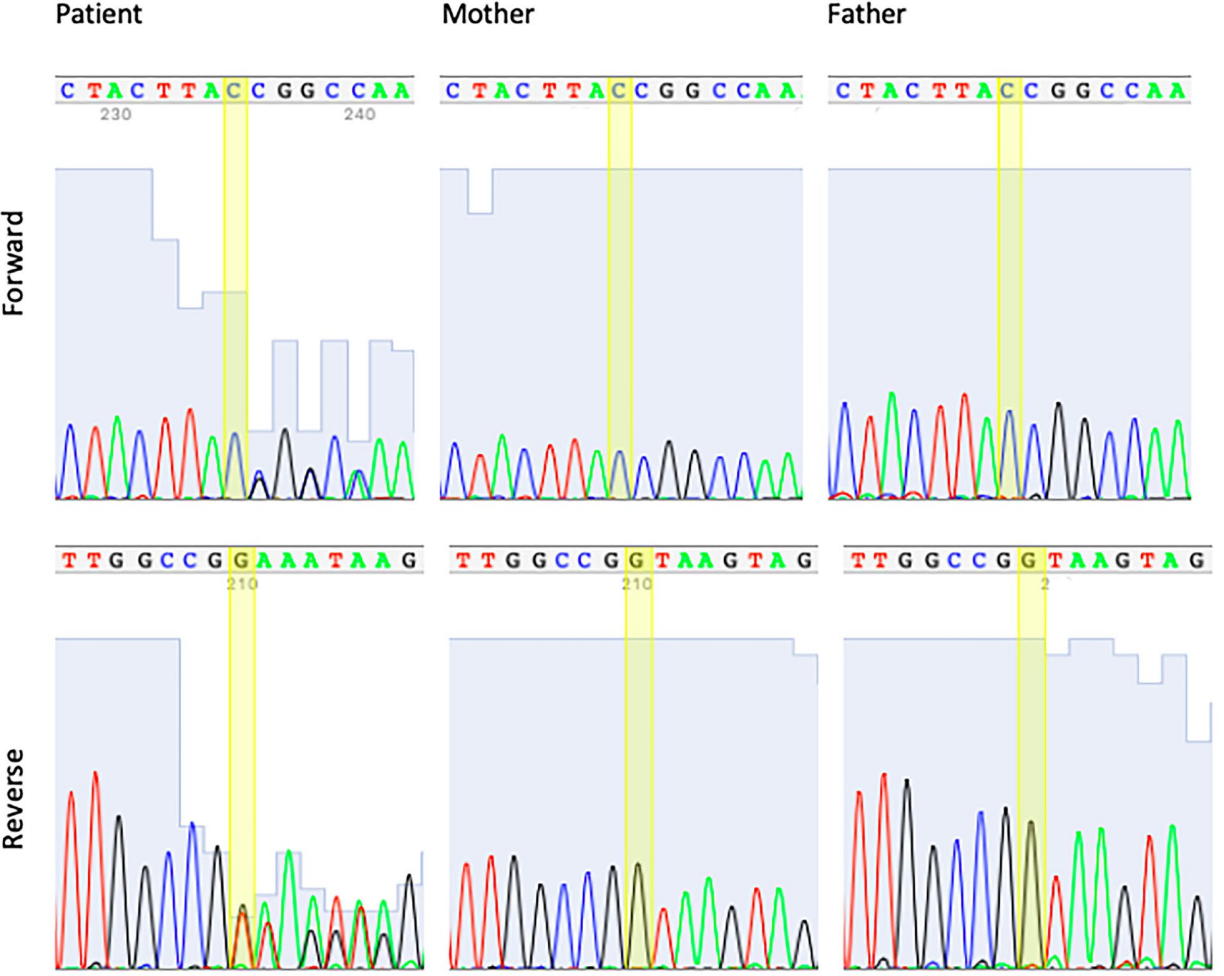
Forward and reverse reads obtained from Sanger Sequencing of the patient and her biological parents

Sanger results indicate that the variant, c.275 + 1del, occurred de novo. Biological parents of the patient have reference alleles in the same position. In addition to the parental verification, mixed and low-quality traces from the heterozygous deletion point in both forward and reverse sequence direction confirm the IGV visualization (Fig. 3).

## Discussion and conclusions

Clinical exome sequencing is considered as a powerful approach for identifying disease-causing variants, even though it has a 25–30% diagnostic yield for patients [37]. Some of these undiagnosed patients already have a pathogenic variant in WES, but it may not be identified in the initial analysis. A recent study showed that 10% of the undiagnosed patients could get a precise diagnosis through the re-analysis of the same WES data, by applying different workflows and with the help of growing knowledge in the literature [38].

Here, we report a patient who had previously unsolved WES data. The in-house variant prioritization workflow was employed to the raw data of the patient. The workflow revealed the novel heterozygous variant c.275 + 1del in the *SLC2A1* gene. To our knowledge, this variant has not been reported in the literature or the available databases. Sanger results indicate that the variant has occurred de novo. After extensive neurometabolic and genetic screen, the patient was diagnosed with GLUT1DS1. Then the patient started on the KD, and her seizures responded to the KD. She has been seizure-free shortly after the initiation of the diet. She also had decreased involuntary movements, her speech became more understandable, and her vocabulary increased after the diet. Thus, this study has also shown that the importance of dietary therapeutic approaches after the investigation of genetic causes behind a disease.

Interestingly, the missense splicing variant c.275 + 1G > A, which is in the same position as our patient, was reported before by Ismayilova et al. [39] (Table 1). The nucleotide sequences on the exon–intron boundaries are usually highly conserved and crucial for the regulation of gene expression [40, 41]. Mutations at these splicing sites may affect the gene expression drastically, which can explain the disease phenotype occurred in both individuals. Although both patients had some phenotypic overlap, including global developmental delay, seizure, signal abnormality in white matter, our patient showed some additional symptoms including microcephaly and several complex movement anomalies such as dystonia and choreoathetosis. However, the previously reported patient has a lower CSF glucose level and CSF/serum ratio. The symptomatic differences between these cases may arise from alternate modifying genetic features and epigenetic factors. The causative mechanisms have to be further investigated to understand the dissimilarity.

**Table 1:** Comparison of reported individuals with a different mutation at the location c.275 + 1 on *SLC2A1*

	This Report, 2020	Ismayilova et al., 2018
Genotype	c.275 + 1del	c.275 + 1G > A
Clinical Findings	Epilepsy, developmental delay, white matter hyperintensities, movement disorders (dystonia and choreoathetosis), microcephaly	Epilepsy, developmental delay, white matter hyperintensities, truncal hypotonia
CSF glucose (mg/dL)	32	21.6
CSF/serum ratio	0.36	0.24

In addition to typical symptoms of GLUT1DS1, our patient also has a hemangioma as an unusual feature. Although the hemangioma pathogenesis is not completely clarified, GLUT1 protein has been used as a selective marker to differentiate hemangioma from other vascular malformations [42, 43]. It is supposed that the expression of GLUT1 in hemangioma tissue is a potential sign of the pathogenicity because the protein is not expressed in the healthy skin tissue [42]. Even though GLUT1 plays a role in both conditions, this occurrence can be coincidental. Further molecular and bioinformatic analyses are needed to establish whether there is a relationship between hemangioma and GLUT1DS1, but, based on our knowledge, these two conditions were not previously reported together.

## Data Availability

The novel variant revealed during the study has been submitted to ClinVar repository with the accession number SCV001548248. The datasets analyzed during the study are available in the NCBI Sequence Read Archive (SRA) with the bioproject accession number PRJNA706171. The Sanger Sequencing data generated in the study has been submitted to NCBI GenBank BankIt with the accession numbers MW883607 and MW883608. Reference sequences used in this study for SLC2A1 are available in the following link (https://www.ncbi.nlm.nih.gov/gene/6513#reference-sequences). Databases used in this study were human hg19 reference genome assembly (http://hgdownload.soe.ucsc.edu/goldenPath/hg19/bigZips/hg19.fa.gz), ClinVar database (https://www.ncbi.nlm.nih.gov/clinvar), dbSNP (https://www.ncbi.nlm.nih.gov/snp/), gnomAD Browser (https://gnomad.broadinstitute.org/).

## Abbreviations

ACMG: American College of Medical Genetics and Genomics
BWA: Burrows-Wheeler Aligner
CADD: Combined Annotation Dependent Depletion
CSF: Cerebrospinal Fluid
GATK: Genome Analysis Toolkit
GLUT1: Glucose Transporter Type 1
GLUT1DS1: GLUT1 Deficiency Syndrome 1
gnomAD: The Genome Aggregation Database
IGV: The Integrative Genomics Viewer
KD: Ketogenic Diet
LP: Lumbar Puncture
M-CAP: Mendelian Clinically Applicable Pathogenicity;
MGI: Mouse Genome Informatics
MRI: Magnetic Resonance Imaging
OMIM: Online Mendelian Inheritance in Man
REVEL: Rare Exome Variant Ensemble Learner
*SLC2A1*: Solute Carrier Family 2, Member 1
WES: Whole Exome Sequencing
